# Mitochondrial Calcium Disorder Affects Early Embryonic Development in Mice through Regulating the ERK/MAPK Pathway

**DOI:** 10.1155/2022/8221361

**Published:** 2022-05-20

**Authors:** Luyao Zhang, Kexiong Liu, Qingrui Zhuan, Zhiqiang Liu, Lin Meng, Xiangwei Fu, Gongxue Jia, Yunpeng Hou

**Affiliations:** ^1^State Key Laboratories of Agrobiotechnology, College of Biological Sciences, China Agricultural University, Beijing, China; ^2^Key Laboratory of Animal Genetics, Breeding and Reproduction, College of Animal Science and Technology, China Agricultural University, Beijing, China; ^3^State Key Laboratory of Sheep Genetic Improvement and Healthy Breeding, Xinjiang Academy of Agricultural and Reclamation Sciences, Shihezi, China; ^4^Key Laboratory of Adaptation and Evolution of Plateau Biota, Northwest Institute of Plateau Biology, Chinese Academy of Sciences, Xining, Qinghai, China; ^5^Qinghai Provincial Key Laboratory of Animal Ecological Genomics, Northwest Institute of Plateau Biology, Chinese Academy of Sciences, Xining, Qinghai, China

## Abstract

The homeostasis of mitochondrial calcium ([Ca^2+^]_mt_) in oocytes plays a critical role in maintaining normal reproductive cellular progress such as meiosis. However, little is known about the association between [Ca^2+^]_mt_ homeostasis and early embryonic development. Two *in vitro* mouse MII oocyte models were established by using a specific agonist or inhibitor targeting mitochondrial calcium uniporters (MCU) to upregulate or downregulate [Ca^2+^]_mt_ concentrations. The imbalance of [Ca^2+^]_mt_ in MII oocytes causes mitochondrial dysfunction and morphological abnormity, leading to an abnormal spindle/chromosome structure. Oocytes in drug-treated groups are less likely to develop into blastocyst during *in vitro* culture. Abnormal [Ca^2+^]_mt_ concentrations in oocytes hindered epigenetic modification and regulated mitogen-activated protein kinase (MAPK) signaling that is associated with gene expression. We also found that MAPK/ERK signaling is regulating DNA methylation in MII oocytes to modulate epigenetic modification. These data provide a new insight into the protective role of [Ca^2+^]_mt_ homeostasis in early embryonic development and also demonstrate a new mechanism of MAPK signaling regulated by [Ca^2+^]_mt_ that influences epigenetic modification.

## 1. Introduction

Female infertility has been a serious problem among women worldwide. Oocyte activation failure is one of the contributing factors to infertility [1, 2] since a properly developed oocyte is decisive for the subsequent fertilization, implantation, and embryonic development [3, 4]. Mitochondrial dysfunction in oocytes caused by obesity, diabetes, or aging has been related to the reduced reproductive capacity [5, 6]. Previous studies have shown that mitochondria exhibit typical maternal hereditary characteristics [7]; meanwhile, their abundant production of adenosine triphosphate (ATP) is required for the reproductive process such as [8, 9].

Mitochondrial calcium homeostasis is associated with oocyte activation disorders when mitochondrial energy production is impaired by oxidative stress or energy deficiency. Through establishing *in vitro* cell models (knocking down the gatekeepers of the [Ca^2+^]_mt_ uniporters Micu1 and Micu2 or mitochondrial Na^+^-Ca^2+^ exchangers in germinal vesicle (GV) oocytes to realize [Ca^2+^]_mt_ overload and knocking down mitochondrial calcium uniporter (MCU) to realize [Ca^2+^]_mt_ defection), our previous studies found that both overload and defection of [Ca^2+^]_mt_ would delay oocyte meiotic maturation. Damaged mitochondrial function and delayed meiotic resumption were also observed in these models [5, 10]. These results altogether highlight the role of [Ca^2+^]_mt_ in oocyte meiosis; in particular, a proper level of [Ca^2+^]_mt_ in oocytes at the GV stage can be a biomarker to assess oocyte quality [11], despite that its contribution to oocyte maturation is still unclear. Therefore, the depth of our understanding for how [Ca^2+^]_mt_ determines the quality of metaphase II (MII) oocytes and regulates early embryonic development requires further expansion.

As protein kinases to specific amino acids (e.g., serine, threonine, and tyrosine), mitogen-activated protein kinase (MAPK) families play an important role in complex cellular programs such as cell proliferation [12]. MAPK families have also been related to spindle assembly regulation and microtubule organization [13–15]. Two studies showed that abnormal MAPK signaling blocks the GV breakdown (GVBD) of both porcine and cattle oocytes [16, 17]. Our previous study showed that the calcium-sensing receptor as a gonadotropin-regulated factor promotes the maturation of porcine oocytes in a MAPK-dependent manner [18]. However, the underlying mechanism of how [Ca^2+^]_mt_ correlates with MAPK signaling to regulate the subsequent development of oocyte is yet poorly explored.

Epigenetic regulation is equally important in regulating oocyte maturation and early embryonic development [19]. Previous studies have shown that aberrant intracellular energy levels can induce changes in epigenetic modifications [20]. The crucial role of DNA methylation in early embryonic development has been elucidated [21] since an abnormal DNA methylated regulation may lead to abnormal development of oocyte and subsequent embryonic cleavage [22]. Although the regulatory role of DNA methylation in embryonic development has been elucidated [23], the relationship between [Ca^2+^]_mt_ and DNA methylation has not been fully determined.

In this study, we established two *in vitro* mouse oocyte models using either an activator or inhibitor targeting [Ca^2+^]_mt_ uniporters: (1) increasing [Ca^2+^]_mt_ by spermine and (2) decreasing [Ca^2+^]_mt_ by Ru360. Our research is aimed at understanding how [Ca^2+^]_mt_ homeostasis determines the competence of oocytes developing into the embryo, as well as correlations among [Ca^2+^]_mt_ homeostasis, MAPK signaling pathway, and epigenetic inheritance.

## 2. Results

### 2.1. Establishment of the [Ca^2+^]_mt_ Disorder Model by the MCU Activator and Inhibitor

**Figure 1:**
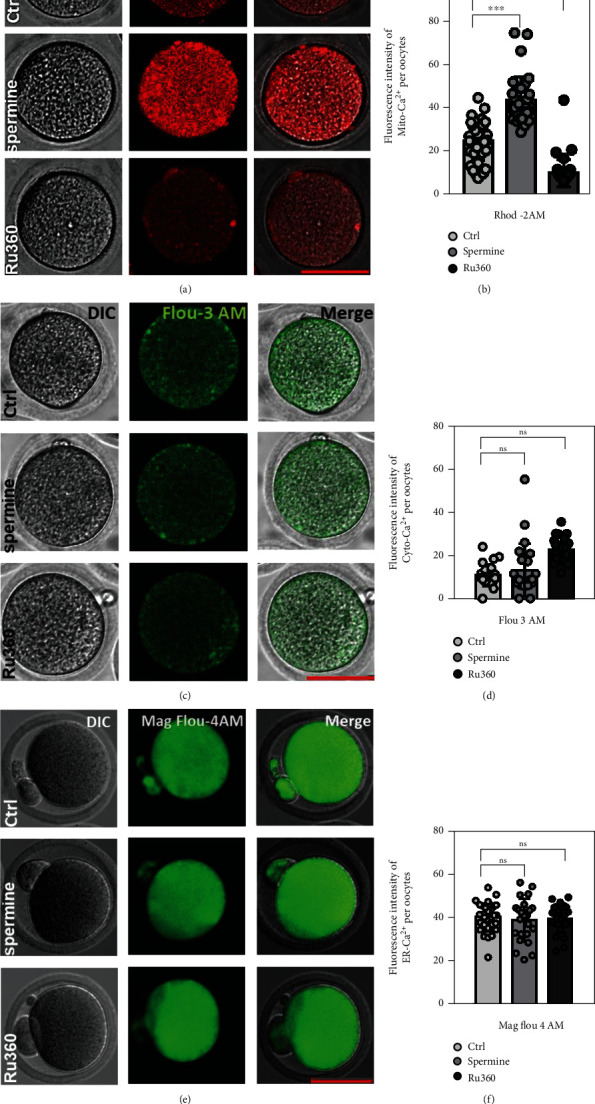
Treating MII oocytes with spermine or Ru360 to establish [Ca^2+^]_mt_ disorder models. (a) Representative images of [Ca^2+^]_mt_ in MII oocytes detected by Rhod-2 AM staining after different treatments. Scale bar, 50 *μ*m. (b) The fluorescence intensity of Rhod-2 AM was counted in control (*n* = 40) and spermine- (*n* = 34) and Ru360-treated (*n* = 41) groups. (c) Representative images of [Ca^2+^]_i_ in MII oocytes detected by Flou-3 AM staining after different treatments. Scale bar, 50 *μ*m. (d) The fluorescence intensity of Flou-3 AM was counted in control (*n* = 24) and spermine- (*n* = 23) and Ru360-treated (*n* = 25) groups. (e) Representative images of [Ca^2+^]_ER_ in MII oocytes detected by Mag Flou-4 AM staining after different treatments. Scale bar, 50 *μ*m. (f) The fluorescence intensity of Mag Flou-4 AM was counted in control (*n* = 32) and spermine- (*n* = 23) and Ru360-treated (*n* = 25) groups. Two-tailed paired Student's *t*-test was used for statistical analyses. Data are shown as mean ± SEM. ^∗∗∗^*P* < 0.001; n.s. indicates nonsignificance (*P* > 0.05); SEM: standard error of the mean.

Despite that previous research has shown that [Ca^2+^]_mt_ disorder led to delayed meiotic maturation and mitochondrial dysfunction in GV oocytes [5, 10], the regulatory role of [Ca^2+^]_mt_ homeostasis in matured oocytes is still unclear. As shown in Figures 1(a) and 1(b), [Ca^2+^]_mt_ levels in MII oocytes markedly increased after spermine treatment, while Ru360-treated MII oocytes showed the opposite (25.33 ± 1.43 for control, 44.32 ± 1.84 for spermine treatment, and 10.33 ± 0.98 for Ru360 treatment; Figures 1(a) and 1(b)). There is no significant difference in [Ca^2+^]_ER_ or [Ca^2+^]_i_ between the control and drug-treated MII oocytes (Figures 1(c) and 1(d)).

### 2.2. [Ca^2+^]_mt_ Disorder Damaged Spindle/Chromosome Structure in MII Oocytes

**Figure 2:**
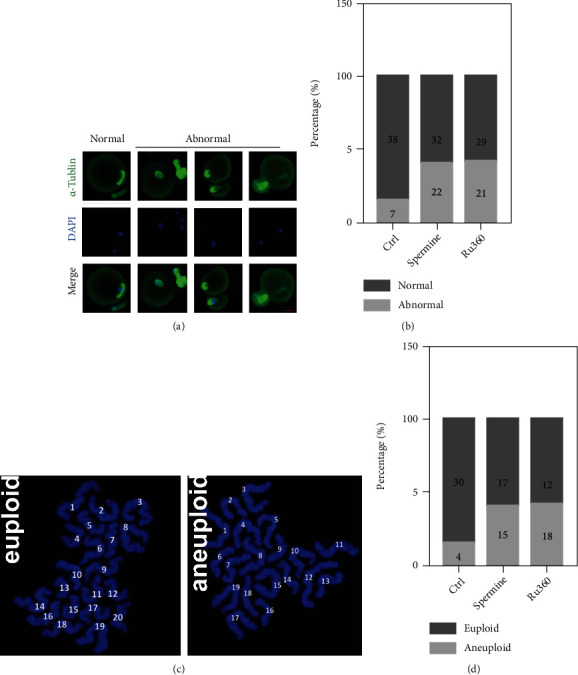
Effect of [Ca^2+^]_mt_ disorder on spindle/chromosome structure in MII oocytes. (a) MII oocytes after different treatments were costained with anti *α*-tubulin antibodies and DAPI to visualize spindles (green) and chromosomes (blue), respectively. Scale bar, 10 *μ*m. (b) Quantification of abnormal spindles for MII oocytes after different treatments (control: *n* = 45; spermine: *n* = 54; and Ru360: *n* = 50). (c) Representative images of euploid and aneuploid oocytes. Chromosome spreading was performed to quantify chromosomes in MII oocytes after different treatments. (d) Quantification of aneuploidy in MII oocytes after different treatments (control: *n* = 34; spermine: *n* = 32; and Ru360: *n* = 30).

[Ca^2+^]_mt_ disorder increased the percentage of spindle defects in MII oocytes (15.6% for control, 40.7% for spermine treatment, and 42% for Ru360 treatment; Figures 2(a) and 2(b)). Aberrant spindle assembly and incorrect chromosome alignment are highly correlated with defective attachment between kinetochores and microtubules, leading to aneuploidy [3]. Normally, the number of chromosomes in mouse MII oocytes is 20 (Figure 2(c)), which is necessary for genomic integrity. However, a higher frequency of aneuploid oocytes that had more or less than 20 univalent was found in spermine- or Ru360-treated oocytes (11.7% for control, 46.9% for spermine treatment, and 60% for Ru360 treatment; Figure 2(d)). Together, these findings indicate that [Ca^2+^]_mt_ homeostasis is required for spindle assembly and nuclear maturation during meiosis.

### 2.3. [Ca^2+^]_mt_ Leads to Mitochondrial Dysfunction in MII Oocytes

**Figure 3:**
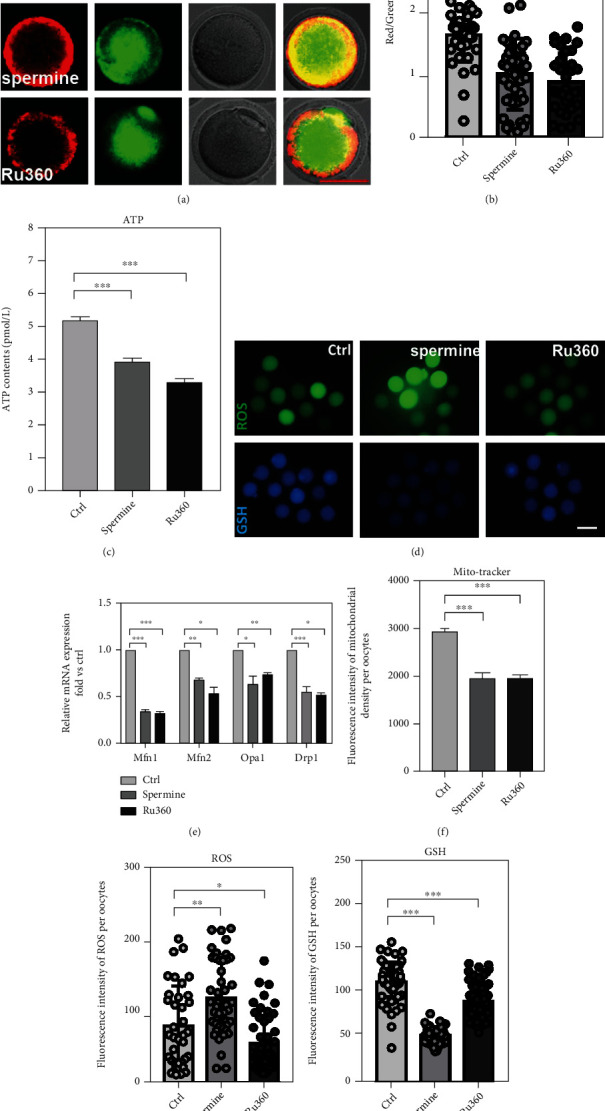
Effect of [Ca^2+^]_mt_ disorder on mitochondrial distribution and function in MII oocytes. (a) Δ*φ*m determined by JC-1 staining in MII oocytes after different treatments. Red indicates high Δ*φ*m, while green indicates low Δ*φ*m. Scale bar: 50 *μ*m. (b) The ratio of red to green fluorescence intensity in MII oocytes after different treatments: control (*n* = 40), spermine (*n* = 45), and Ru360 (*n* = 36). (c) ATP concentrations in individual MII oocytes after different treatments (*n* = 30 for each group). (d) Representative images of ROS levels detected by DCFH staining and GSH levels detected by DTNB staining in MII oocytes after different treatments. Scale bar, 50 *μ*m. (e) Expression levels of *Opa1*, *Mfn1*, *Mfn2*, and *Drp1* in MII oocytes after different treatments (*n* = 30 for each group). (f) The fluorescence intensity of MitoTracker in MII oocytes after different treatments: control (*n* = 30), spermine (*n* = 25), and Ru360 (*n* = 29). (g) The fluorescence intensity of ROS in MII oocytes after different treatments: control (*n* = 36), spermine (*n* = 46), and Ru360 (*n* = 49). (h) The fluorescence intensity of GSH in MII oocytes after different treatments: control (*n* = 51), spermine (*n* = 60), and Ru360 (*n* = 61). Two-tailed paired Student's *t*-test was used for statistical analyses. Data are shown as mean ± SEM. ^∗^*P* < 0.05, ^∗∗^*P* < 0.01, and ^∗∗∗^*P* < 0.001.

As expected, levels of MMP (1.59 ± 6.9% for control, 0.98 ± 8.4% for spermine treatment, and 0.86 ± 7.7% for Ru360 treatment; Figures 3(a) and 3(b)) and ATP (5.20 ± 11.63% pmol for control, 3.93 ± 14.13% pmol for spermine treatment, and 3.31 ± 9.44% pmol for Ru360 treatment; Figure 3(c)) were both decreased in drug-treated groups compared to controls. Moreover, mitochondrial dysfunction might lead to oxidative stress [5, 24]. The level of reactive oxygen species (ROS) increased (79.54 ± 9.32 for control, 118.79 ± 7.66 for spermine treatment, and 54.97 ± 5.65 for Ru360 treatment; Figure 3(g)) while the level of GSH decreased in oocytes after drug treatments (114.04 ± 3.26 for control, 52.90 ± 1.13 for spermine treatment, and 92.13 ± 2.82 for Ru360 treatment; Figure 3(h)), which coincided with our hypothesis. Additionally, mRNA levels of genes regulating mitochondrial fusion (*Opa1*, *Mfn1*, and *Mfn2*) and fission (*Drp1*) were abnormally expressed in spermine- or Ru360-treated oocytes (Figure 3(e)). Lastly, mitochondrial density in oocytes decreased significantly after drug treatments (2934.60 ± 135.80 for control, 1950.46 ± 89.11 for spermine treatment, and 1952.48 ± 111.72 for Ru360 treatment; Figure 3(f)). Collectively, these data suggested that mitochondrial function and dynamics are impaired in MII oocytes with [Ca^2+^]_mt_ disorder.

### 2.4. Effect of [Ca^2+^]_mt_ Disorder on Early Embryonic Development and Epigenetic Modifications of Oocytes

**Figure 4:**
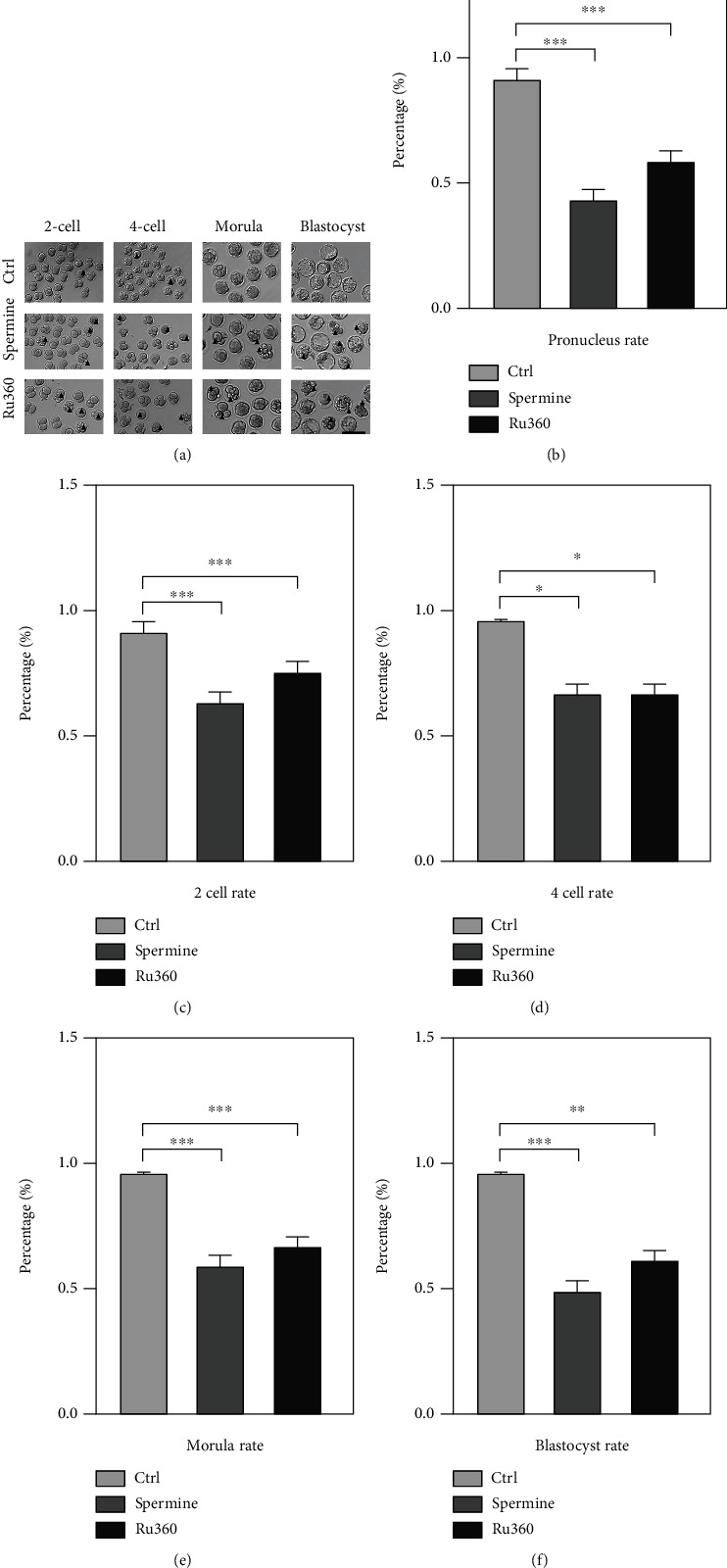
Effect of [Ca^2+^]_mt_ disorder on early embryonic development of oocytes. (a) Representative images of early embryos developed from MII oocytes in control and spermine- and Ru360-treated groups. Scale bar, 50 *μ*m. (b) The pronucleus rate of the embryo after different treatments: control (*n* = 112), spermine (*n* = 132), and Ru360 (*n* = 177). (c) The rate of 2-cell embryos after different treatments: control (*n* = 107), spermine (*n* = 162), and Ru360 (*n* = 147). (d) The rate of 4-cell embryos after different treatments: control (*n* = 105), spermine (*n* = 161), and Ru360 (*n* = 115). (e) The rate of morula formation after different treatments: control (*n* = 106), spermine (*n* = 126), and Ru360 (*n* = 122). (f) The rate of blastocyst formation after different treatments: control (*n* = 97), spermine (*n* = 120), and Ru360 (*n* = 126). Two-tailed paired Student's *t*-test was used for statistical analyses. Data are shown as mean ± SEM. ^∗^*P* < 0.05, ^∗∗^*P* < 0.01, and ^∗∗∗^*P* < 0.001.

**Figure 5:**
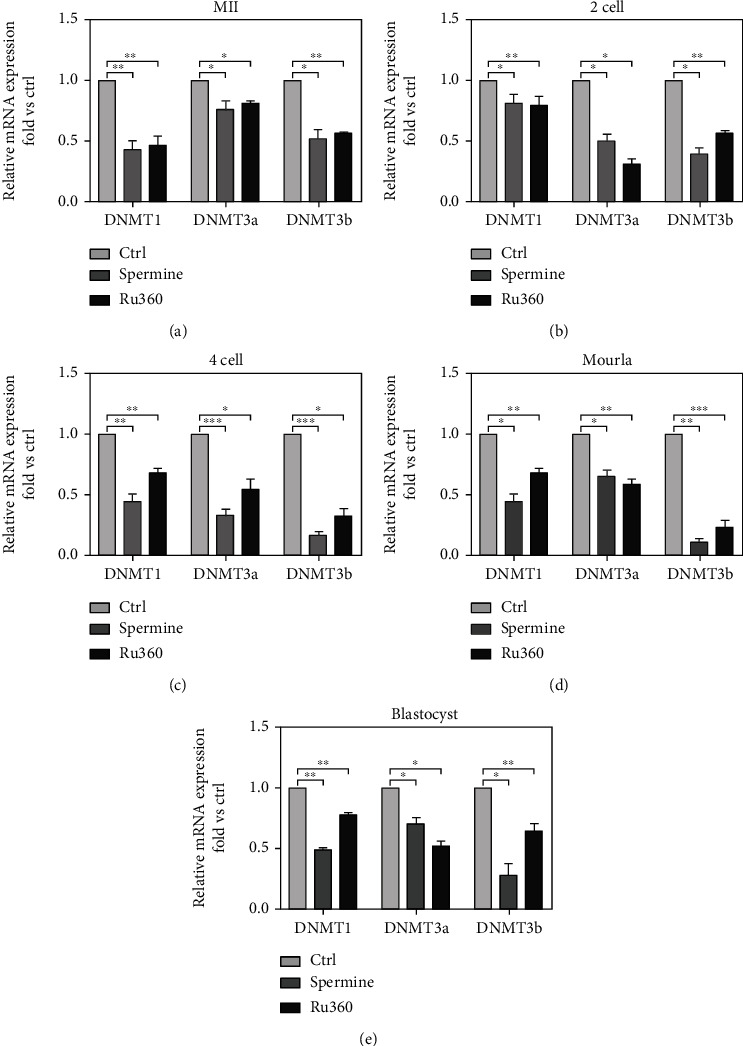
mRNA level of *DNMT1*, *DNMT3a*, and *DNMT3b* in MII oocytes with [Ca^2+^]_mt_ disorder and embryos in different stages. mRNA levels of genes encoding DNMT1, DNMT3a, and DNMT3b after different treatments in (a) MII oocytes (*n* = 30 for each group), (b) 2-cell embryo (*n* = 25 for each group), (c) 4-cell embryo (*n* = 25 for each group), (d) morulae (*n* = 20 for each group), and (e) blastocysts (*n* = 15 for each group). Two-tailed paired Student's *t*-test was used for statistical analyses. Data are shown as mean ± SEM. ^∗^*P* < 0.05, ^∗∗^*P* < 0.01, and ^∗∗∗^*P* < 0.001.

We then further study the competence of MII oocytes developing into the embryo after drug-induced [Ca^2+^]_mt_ disorder using parthenoactivation. It showed that most MII oocytes could be activated and then developed into 2-cell embryos in the control group, whereas drug-treated MII oocytes showed a dramatically lower rate of pronucleus formation compared to controls (95.01% ± 4.71% for control, 46.84% ± 2.90% for spermine treatment, and 56.93% ± 4.10% for Ru360 treatment; Figures 4(a) and 4(b)). Likewise, lower rates of 2-cell embryos (94.27% ± 2.87% for control, 75.19% ± 2.16% for spermine treatment, and 82.97% ± 2.18% for Ru360 treatment; Figure 4(c)), 4-cell embryos (95.31% ± 0.58% for control, 75.96% ± 4.74% for spermine treatment, and 73.67% ± 5.37% for Ru360 treatment; Figure 4(d)), morula rate (96.30% ± 1.92% for control, 65.82% ± 2.74% for spermine treatment, and 74.52% ± 0.72% for Ru360 treatment; Figure 4(e)), and blastocyst (92.43% ± 2.44% for control, 48.71% ± 2.09% for spermine treatment, and 54.86% ± 4.06% for Ru360 treatment; Figure 4(f)) in drug-treated groups were observed. The expression levels of *DNMT1*, *DNMT3a*, and *DNMT3b* at different embryonic stages were significantly lower in drug-treated oocytes compared to those in the control group (Figure 5(a)–5(e)). These results suggest that [Ca^2+^]_mt_ disorder impaired the competence of MII oocytes developing into embryos, along with changed epigenetic modifications.

### 2.5. [Ca^2+^]_mt_ Disorder Affects Early Embryonic Development in Mice through the MAPK/ERK Pathway

**Figure 6:**
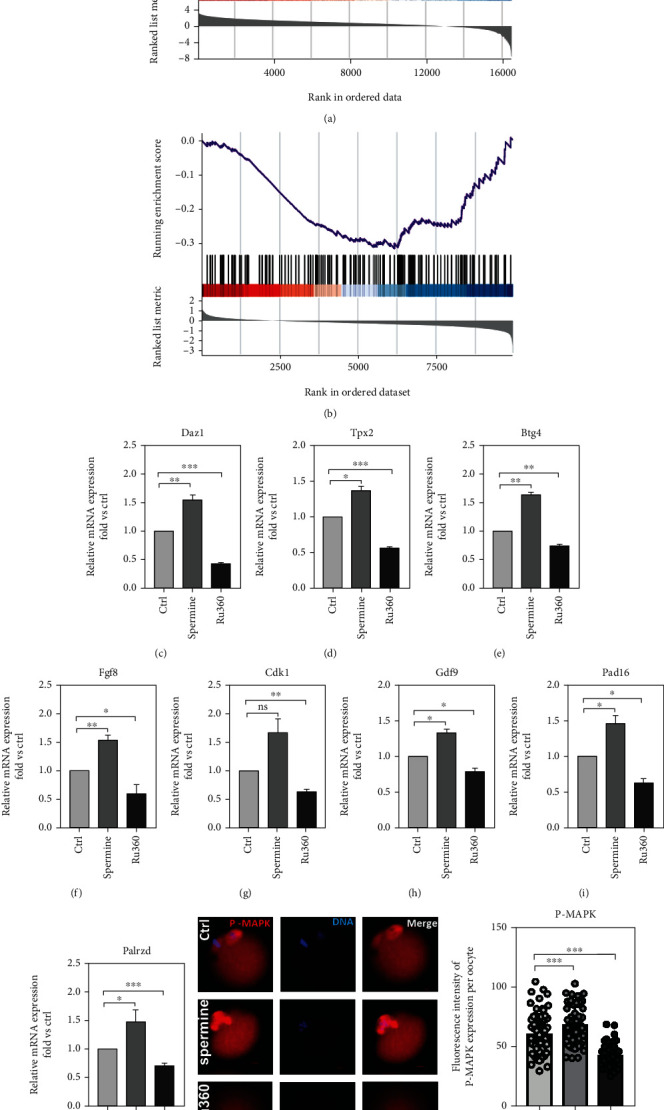
[Ca^2+^]_mt_ disorder affects early embryonic development in mice through moderating the ERK/MAPK signaling pathway. (a, b) GSEA indicated differences of DEGs in the control compared to [Ca^2+^]_mt_ overload or decline group enriched in the MAPK signaling pathway. |NES| > 1 and FDR < 0.1. (c–j) mRNA level of genes related to the ERK/MAPK signaling pathway in MII stage oocytes (*n* = 30 for each group). (k) The subcellular localization and expression of p-MAPK (red) in MII oocytes after different treatments. Scale bar, 50 *μ*m. (l) The relative levels of p-MAPK in MII oocytes after different treatments: control (*n* = 55), spermine (*n* = 58), and Ru360 (*n* = 66). Two-tailed paired Student's *t*-test was used for statistical analyses. Data are shown as mean ± SEM. ^∗^*P* < 0.05, ^∗∗^*P* < 0.01, and ^∗∗∗^*P* < 0.001.

The effects of [Ca^2+^]_mt_ disorder on mitochondrial function and early embryo development led us to study the underlying mechanisms. Previous studies using *in vitro* GV oocyte models showed that *Dazl*, *Tpx2*, *Btg4*, *Fgf8*, *Cdk1*, *Gdf9*, *Padl6*, and *Palrzd*in MAPK signaling were upregulated after [Ca^2+^]_mt_ defection and downregulated after [Ca^2+^]_mt_ overload, respectively [5, 10] (Figures 6(a) and 6(b)). Here, we found that the mRNA levels of those key genes related to the MAPK/ERK signaling pathway were upregulated in spermine-treated oocytes and downregulated in those Ru360-treated counterparts (Figures 6(c)–6(k)). Also, an increased protein level of phosphorylated MAPK (p-MAPK) in MII oocytes was observed after spermine treatment, whereas it was decreased in the Ru360 treatment group (42.65 ± 1.24 for control, 68.48 ± 2.09 for spermine treatment, and 60.41 ± 2.17 for Ru360 treatment; Figures 6(l) and 6(m)). Notably, the addition of U0126 (inhibitor targeting MAPK) and curcumin (activator targeting MAPK) could neutralize detrimental effects on the embryonic development of oocytes induced by treatments with spermine and Ru360, respectively (Table 1). Above results suggest that the developmental deficiency of oocytes with [Ca^2+^]_mt_ disorder is a result of a perturbed MAPK/ERK signaling pathway.

### 2.6. Abnormal MAPK/ERK Signaling May Damage Epigenetic Modifications of Oocytes

**Figure 7:**
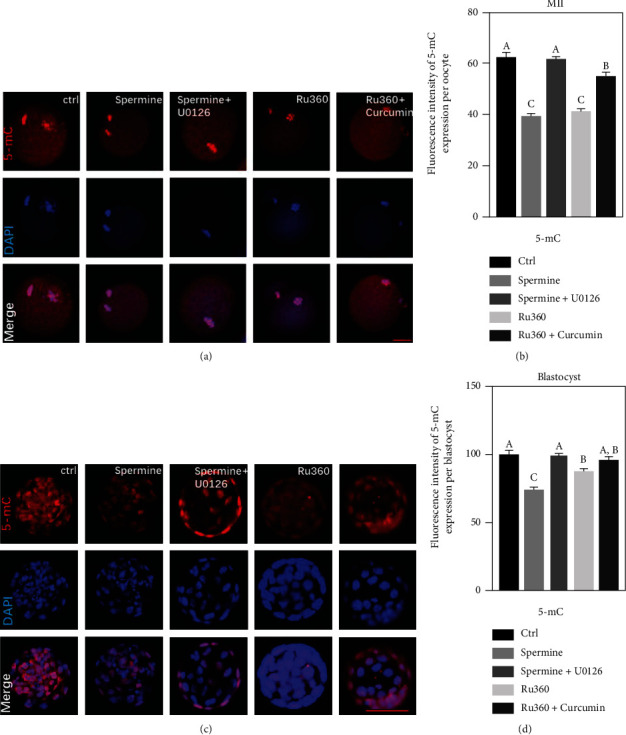
Abnormal ERK/MAPK signaling pathway may damage epigenetic modifications of oocytes. (a) The subcellular localization and expression of 5-mC (red) in MII oocytes after different treatments. DAPI staining is shown in blue. Scale bar, 10 *μ*m. (b) The relative levels of 5-mC in MII oocytes after different treatments: control (*n* = 48), spermine (*n* = 45), spermine+U0126 (*n* = 56), Ru360 (*n* = 60), and Ru360+curcumin (*n* = 36). (c) The subcellular localization and expression of 5-mC (red) in blastocysts developed from MII oocytes after different treatments. DAPI staining is shown in blue. Scale bar, 50 *μ*m. (d) The relative levels of 5-mC in blastocysts developed from MII oocytes after different treatments: control (*n* = 48), spermine treatment (*n* = 30), spermine+U0126 treatment (*n* = 42), Ru360 treatment (*n* = 44), and Ru360+curcumin treatment (*n* = 37). One-way ANOVAs were used for statistical analyses. Different superscript letters (A–C) indicate significant differences of measurements in the same column (*P* < 0.05).

**Figure 8:**
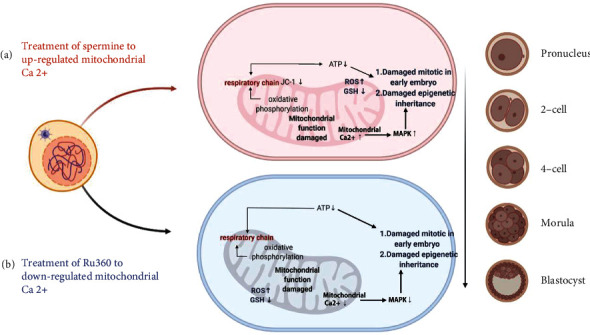
Model for [Ca^2+^]_mt_ disorder and abnormal MAPK activity in MII oocytes and early blastocysts. Oocytes with damaged mitochondrial function show [Ca^2+^]_mt_ disorder, followed by a decline in cytosolic ATP level. In consequence, MAPK signaling is upregulated by [Ca^2+^]_mt_ overload or downregulated by [Ca^2+^]_mt_ defection. Overactivation and suppression of MAPK in GV oocytes both hinder the resumption of meiosis. In addition, epigenetic inheritance is damaged by ATP defection and abnormal MAPK activities, leading to an impaired early embryonic development. These observations implicate that [Ca^2+^]_mt_ homeostasis is critical for early embryonic development in mice.

Aberrant energy supply and oxidative stress are highly correlated with epigenetic modifications, leading to deficiency of embryonic development [25]. DNA methylation studies on drug-treated oocytes showed that 5-mC levels in MII oocytes decreased after both drug treatments compared to those in the control (61.69 ± 1.93 for control, 38.77 ± 1.11 for spermine treatment, and 40.61 ± 1.15 for Ru360 treatment; Figures 7(a) and 7(b)); a similar trend was also observed in blastocysts (100.31 ± 2.20 for control, 81.93 ± 3.12 for spermine treatment, and 90.42 ± 2.55 for Ru360 treatment; Figures 7(c) and 7(d)). To further understand relationships among the MAPK/ERK pathway, [Ca^2+^]_mt_ homeostasis, and epigenetic inheritance, the 5-mC expression levels in spermine-treated oocytes or blastocysts with additional treatment with U0126 and those Ru360-treated counterparts with curcumin were studied. Both U0126 and curcumin neutralized the decreased expression of 5-mC in MII oocytes (61.01 ± 1.07 for spermine+U0126 treatment, 54.29 ± 2.35 for Ru360+curcumin treatment; Figure 7(b)) and blastocysts (99.73 ± 2.71 for spermine+U0126 treatment, 95.62 ± 2.65 for Ru360+curcumin treatment; Figure 7(d)) after [Ca^2+^]_mt_ disorders induced by drug treatments, suggesting that [Ca^2+^]_mt_ disorders also cause changes in epigenetic modification in oocytes (Figure 8).

## 3. Discussion

Obese, diabetic, and aging women having lower rate of oocyte activation, altered preimplantation embryo development, and even lower pregnancy rates have been highlighted [26–29], and these disorders have been related to the mitochondrial dysfunction in oocytes [5], because the ATP production and oxidative phosphorylation are essential for reproductive cellular processes including meiotic maturation and postimplantation development [30]. Our previous studies highlight the significance of [Ca^2+^]_mt_ balance in GV oocytes since low levels of [Ca^2+^]_mt_ display a higher frequency of spindle defects in meiosis and affect oocyte maturation, while [Ca^2+^]_mt_ overload induces a delay of meiosis maturation, mitochondrial dysfunction, and oxidative stress [5]. In this study, spermine and Ru360 were used as an agonist or inhibitor of MCU to study mitochondrial function and developmental competence in MII oocytes undergoing [Ca^2+^]_mt_ imbalance. Firstly, we compared levels of [Ca^2+^]_i_, [Ca^2+^]_ER_, and [Ca^2+^]_mt_ between control and drug-treated groups. The level of [Ca^2+^]_mt_ was upregulated in the spermine-treated group and downregulated in the Ru360-treated group (Figures 1(a) and 1(b)) while there is no significant difference in [Ca^2+^]_i_ and [Ca^2+^]_ER_ (Figure 1(c)–1(f)), indicating that spermine- or Ru360-treated MII oocytes are available in *in vitro* models to study the impact of [Ca^2+^]_mt_ disorder.

We further found that the spindle/chromosome structure was damaged in drug-treated MII oocytes (Figures 2(a)–2(d)), indicating that oocytes are in poor quality, also supporting our previous studies that the depletion of MCU markedly disrupts spindle formation during oocyte meiosis [10]. The generation of ATP is one of the basic functions of mitochondria, and its cytoplasmic level reflects the quality of matured oocytes [31, 32]. We then studied the effects of spermine and Ru360 on mitochondrial function (e.g., mitochondrial membrane potential and ATP levels in MII oocytes). Both mitochondrial membrane potential and ATP levels in MII oocytes were decreased after treatments with spermine and Ru360. In addition, changes in mitochondrial morphology were observed in drug-treated groups including decreased mitochondrial densities and downregulated genes which are related to mitochondrial morphodynamics (Figures 3(e) and 3(f)). Consistently, defection in [Ca^2+^]_mt_ homeostasis damaged the mitochondrial function of MII oocytes, followed by impaired competence developing into the embryo (Figure 4).

The [Ca^2+^]_mt_ homeostasis in meiotic maturation of oocytes has been substantiated to be dependent on MAPK signaling pathways. The MAPK family is involved in regulating meiosis recovery in the follicle before ovulation as a signaling molecule; to be more specific, Mapk3/1 (also commonly known as ERK1/2) plays a role in gonadotropin-induced signal transduction, and its activation in cumulus cells is critical for gonadotropin-induced GVBD and granulosa cell proliferation [33]. In this study, MII oocytes display an aberrant expression of p-MAPK after treatment with spermine or RU360, and their inability to develop into the early embryo can be neutralized by extra treatment by either U0126 or curcumin, respectively (Figures 6(l) and 6(m) and Table 1). Collectively, oocytes with [Ca^2+^]_mt_ disorder having a lower rate of embryonic formation (Figures 4(a)–4(f)) are possibly caused by the perturbed MAPK/ERK signaling pathway.

Epigenetic inheritance is a key factor in mediating oocyte maturation and early embryonic development [34, 35]. Previous studies have shown that both ATP and ROS are concomitantly produced in the mitochondria via the tricarboxylic acid cycle, of which disturbance results in aberrant epigenetic modifications [36]. Moreover, the modification of DNA plays roles in oogenesis and early embryogenesis [37, 38]. In this study, disruption of global genomic DNA methylation and altered expression levels of individual genes related to DNA methylation in MII oocytes were observed after treatments with spermine or Ru360 (Figures 5(a)–5(e)).

The involvement of the MAPK/ERK signaling pathway in epigenetic regulation in the mouse brain is observed [39]. Whether the level of [Ca^2+^]_mt_ in oocytes is related to epigenetic modifications regulated by the activation of the MAPK signaling pathway remains unknown. Here, we observed a defective expression of 5-mC in drug-treated groups, while these epigenetic abnormalities can be reversed by U0126 or curcumin (Table 1), indicating that [Ca^2+^]_mt_ disorder affects early embryonic development through the MAPK/ERK signaling pathway, of which overactivation or inhibition compromises the DNA methylation (Figure 8). In brief, for MII oocytes, a proper physiological level of [Ca^2+^]_mt_ is critical for its maturation and competence of developing into the embryo.

In summary, our study highlights the role of [Ca^2+^]_mt_ in maintaining both mitochondrial function and competence to develop into the embryo for oocytes, which is fulfilled by mediating the MAPK/ERK signaling pathway and epigenetic modifications. Although the DNA methylation in oocytes with [Ca^2+^]_mt_ disorder has been investigated in the current study, other epigenetic modifications such as histone acetylation await further investigation.

## 4. Conclusion

Our research using oocytes with [Ca^2+^]_mt_ disorder as *in vitro* models proved that [Ca^2+^]_mt_ plays a role in maintaining mitochondrial function and regulating epigenetic modification for oocytes. In addition, epigenetic inheritance can be damaged by ATP defection and the perturbed MAPK/ERK signaling pathway, leading to an impaired competence of MII oocytes to develop into the embryo.

## 5. Methods and Materials

### 5.1. Ethics Statement

All chemicals and medicines were purchased from the Sigma Chemical Co. (St. Louis, MO, USA) unless otherwise described. Seven-week-old CD-1® (ICR) mice were bought from the Beijing Vital River Experimental Animals Centre (Beijing, China) and were raised at the Department of Animal Experiments under standard housing conditions. The Laboratory Animal Care and Use Committee of the Institute of Zoology approved this research (AW01040202-1).

### 5.2. Oocyte Collection and Culture

Germinal vesicle (GV) stage oocytes were collected from the 3-week-old ICR mice. 5 IU of pregnant mare serum gonadotropin (PMSG, Ningbo Second Hormone Factory, Ningbo, China) was injected into the mice 46-48 hours before all experiments. GV stage oocytes were released from the fully grown follicles into prewarmed M2 medium supplemented with 2.5 *μ*M milrinone, and cumulus cells were removed by repeated pipetting. After microinjection or any specific treatment, oocytes were thoroughly washed with DPBS and cultured in M16 medium under mineral oil at 37°C in a 5% CO_2_ atmosphere incubator during the GV to MII stages.

### 5.3. Parthenoactivation of MII Oocytes

The activation medium was calcium- (Ca^2+^-) free HTF complemented with 10 mM strontium chloride (SrCl_2_) and 5 *μ*g/mL cytochalasin B [40]. After being thoroughly washed in activation medium for three times, MII oocytes were activated in activation medium for 2.5 hours and then in regular HTF without SrCl_2_ for 3.5 hours at 37°C with 5% CO_2_. Oocytes were then transferred from activation medium to KSOM plus (+) amino acids (KSOM/AA) medium (EmbryoMax® KSOM + AA with D-glucose and phenol red, EMD Millipore, Billerica, MA, USA). Development of embryos to the 2-cell, 4-cell, morula, and blastocyst stages was assessed 24, 48, 96, and 120 hours, respectively, after the start of initial culture in KSOM/AA medium.

### 5.4. Immunofluorescence

Mouse MII oocytes were fixed in 4% (*w*/*v*) paraformaldehyde for 40 minutes at room temperature and then washed three times (10 minutes each) in washing buffer (PBS containing 0.01% Triton X-100 and 0.1% Tween-20). The oocytes were then permeated in 1% Triton X-100/PBS at room temperature for 1 hour and washed three times (10 minutes each) in washing buffer. The oocytes were then blocked with blocking buffer (1% BSA/PHEM with 100 mM glycine) for 1 hour at 37°C. The oocytes were incubated at 4°C overnight with a primary antibody (anti-*α*-tubulin 1 : 8,000) diluted in blocking buffer. After washing three times with washing buffer for 5 minutes each, the oocytes were incubated at 37°C for 1 hour with a corresponding second antibody (1 : 100 dilutions, CW Biotech) and washed four times in washing buffer for 10 minutes each. Finally, DNA was stained with 4′6-diamidino-2-phenylindole (DAPI, Vector Laboratories Inc., Burlingame, CA, USA). The oocytes were then expanded on glass slides and examined by confocal laser scanning microscopy (FLUOVIEW FV1000, Olympus, Tokyo, Japan) using the FLUOVIEW Viewer (Olympus, Tokyo, Japan). The excitation lasers were set at 488 nm, and emission channels of 520 nm were used for green fluorescence detection.

For 5-methyl cytosine (5-mC) staining, washed oocytes were denatured by 2 N hydrochloric acid (HCl) for 30 minutes at room temperature first and then blocked in blocking buffer for 1 hour. After washing for three times by washing buffer, oocytes were incubated with a mouse anti-5-mC-FITC antibody (1 : 300, Abcam) at 4°C overnight. DNA was then stained with 4′6-diamidino-2-phenylindole (DAPI, Vector Laboratories Inc., Burlingame, CA, USA). Finally, the samples were mounted on glass slides and examined with a confocal laser scanning microscope (FLUOVIEW FV1000, Olympus, Tokyo, Japan) by using the FLUOVIEW Viewer (Olympus, Tokyo, Japan). The excitation lasers were set as 488 nm. The fluorescence intensity of a single oocyte was determined by using EZ-C1 Free-Viewer (Nikon, Tokyo, Japan).

### 5.5. Quantification of [Ca^2+^]_mt_, [Ca^2+^]_i_, and [Ca^2+^]_ER_

Levels of [Ca^2+^]_mt_, [Ca^2+^]_i_, and [Ca^2+^]_ER_ were measured using Rhod-2AM, Flou-3 AM, and Mag Flou-4 AM (Invitrogen/Molecular Probes, Carlsbad, CA, U.S.) staining, respectively, according to the manufacturer's instructions. In brief, the zona pellucida was removed using 0.5% pronase E for 5 minutes. For determining [Ca^2+^]_mt_, pronase E-treated oocytes were incubated in M2 medium complemented with 5 *μ*M Rhod-2AM for 25 minutes. After staining, oocytes were thoroughly washed with DPBS at least three times, followed by incubation in M2 medium free Rhod-2AM at 37°C under a 5% CO_2_ atmosphere for 30 minutes. For determining [Ca^2+^]_i_ and [Ca^2+^]_ER_, pronase E-treated oocytes were incubated in maturation medium complemented with either 5 *μ*M Flou-3 AM for 40 minutes or 5 *μ*M Mag Flou-4 AM for 20 minutes. After that, oocytes were washed with DPBS for three times. Oocytes stained with different dyes were subsequently observed by confocal laser scanning microscopy (Nikon A1R, Tokyo, Japan) and quantified using a NIS-Elements AR (Nikon Instruments, Tokyo, Japan).

### 5.6. Investigation of Mitochondrial Density

Mitochondrial density was quantified by the mitochondrial reactive dye MitoTracker (Green) (Beyotime Institute of Biotechnology, China). Oocytes were placed into M2 medium complemented with 5 *μ*M MitoTracker (Green) for 30 minutes and thoroughly washed with DPBS for three times. Confocal laser scanning microscopy (Nikon A1R, Tokyo, Japan) was used to observe the oocytes, and an NIS-Elements AR (Nikon Instruments, Tokyo, Japan) was used to quantify mitochondria.

### 5.7. Measurement of Intracellular Levels of ROS and GSH

Intracellular levels of ROS and GSH were measured as described previously [41]. In brief, oocytes were incubated in M2 medium supplemented with either 1 mmol/L 2′,7′-dichlorodihydrofluorescein diacetate (H2DCFDA) or 10 *μ*mol/L 4-chloromethyl-6,8-difluoro-7-hydroxycoumarin (Cell-Tracker Blue) for 30 minutes at 37°C and washed with DPBS for three times afterwards. The fluorescence was measured under an epifluorescence microscope with a filter at 460 nm excitation for ROS and 370 nm excitation for GSH (DP72, Olympus, Tokyo, Japan). Finally, the fluorescence intensity of ROS and GSH was determined by using EZ-C1 Free-Viewer (Nikon, Tokyo, Japan).

### 5.8. Quantification of Mitochondrial Membrane Potentials

A mitochondrial membrane potential assay kit (JC-1 dye, Beyotime Institute of Biotechnology, China) was used to measure mitochondrial membrane potentials (Δ*φ*m). Oocytes were stained with a working solution containing 10 *μ*M JC-1 at 37°C in a 5% CO_2_ atmosphere for 20 minutes, after which they were washed with DPBS to remove the surface fluorescence, followed by observation under a fluorescence microscope (Olympus IX73). Red fluorescence indicates activated mitochondria (J-aggregates), while green fluorescence indicates less activated mitochondria (J-monomers), and the ratio of J-aggregates to J-monomers is calculated as the Δ*φ*m value.

### 5.9. ATP Content Assays

The ATP content in each oocyte was measured using the Enhanced ATP Assay Kit S0027 (Beyotime Institute of Biotechnology, China) according to the manufacturer's instructions. Different ATP standards were prepared, ranging from 0 to 40 pmol ATP. Oocytes were then treated with 20 *μ*M of lysis buffer within a 0.2 mL RNA-free centrifuge tube, and lysed cells were centrifuged for 5 minutes at 4°C and 12,000 × *g*. All steps were conducted on ice unless otherwise stated. ATP detecting solution was then added to 96-well plates and was left at room temperature for 3 to 5 minutes. Standard solutions and ATP detection diluents were then added into each well. Samples were also added to each well, and the luminescence signals were immediately calculated with a luminometer (Infinite F200; Tecan). The ATP content of the samples was then calculated based on the standard curves. Total ATP levels were divided by the number of oocytes in each sample to calculate the mean ATP content per oocyte (pmol/oocyte).

### 5.10. Quantitative PCR

Total RNA was extracted using a RNeasy microRNA isolation kit (Qiagen, Valencia, CA, U.S.) following the manufacturer's instructions. Samples were treated with DNase I, and then, Transcript-Uni Cell was used for cDNA synthesis. A quantitative PCR supermix was used for the assays (Transgene Biotech, Beijing, China). RNA concentrations were measured using a Nanodrop 2000 Spectrophotometer (Biolab, Scoresby, Victoria, Australia) at a wavelength of 260 nm. Samples for subsequent analyses were only used if their 260 : 280 nm absorbance ratios were >1.8. Quantitative- and reverse transcription-PCR assays were performed with an ABI 7500 real-time PCR instrument and a Fast 96-well Thermal Cycler (Applied Biosystems, Foster City, CA, U.S.), respectively. Three replicates were conducted for all assays. The relative expression of genes was calculated by the comparative threshold cycle method as 2^−ΔΔCt^. The primers used for the amplification assays are shown in Table 2.

### 5.11. Statistical Analysis

Each experiment was repeated at least three times. A representative image of each experiment is shown. All data were analyzed using Student's *t*-test or one-way analysis of variance (ANOVA) examined by Duncan's multiple range test in SPSS software (IBM, Chicago, IL, USA). Data are expressed as the mean ± SEM.

## Figures and Tables

**Table 1:** Effect of U0126 or curcumin addition on recovering early embryonic development of spermine or Ru360 treatment.

Groups	No. of oocyte culture	Cleavage (%, mean ± SEM)	Blastocyst (%, mean ± SEM)
Ctrl	145	139 (95.7667 ± 0.71259)^a^	99 (88.4667 ± 1.38604)^a^
Spermine	116	74 (63.8333 ± 1.67465)^c^	57 (41.9000 ± 4.30968)^c^
Spermine+U0126	112	99 (90.2000 ± 0.85440)^b^	86 (81.3500 ± 0.75000)^a^
Ru360	108	56 (51.4323 ± 2.14332)^c^	45 (46.3144 ± 3.5439)^c^
Ru360+curcumin	134	105 (80.4553 ± 2.21067)^b^	93 (75.3498 ± 5.33340)^b^

Different superscript letters (a–c) represent a significant difference in the same column (*P* < 0.05). SEM: standard error of the mean.

**Table 2:** Oligonucleotide primer sequences used for quantitative real-time PCR.

Gene	Primer sequence (5′-3′)	Product size (bp)	GenBank accession number or reference
*Btg4*	F:AACCTTTGCACTAAAGCTGATGA R: AGCCCTTTCTAAAACAGGGTCT	142	NM_019493
*Cdk1*	F: AGAAGGTACTTACGGTGTGGT R: GAGAGATTTCCCGAATTGCAGT	128	NM_007659
*Dazl*	F: ATGTCTGCCACAACTTCTGAG R: CTGATTTCGGTTTCATCCATCCT	170	NM_010021
*Dnmt3a*	F: GAGGGAACTGAGACCCCAC R: CTGGAAGGTGAGTCTTGGCA	216	NM_007872
*Dnmt3b*	F:CGTTAATGGGAACTTCAGTGACC R: CTGCGTGTAATTCAGAAGGCT	169	NM_001122997
*Drp1*	F: CAAGGTTTTCTCGCCCAACG R: CTGCCCTTACCATCTGGATCTA	234	NM_001025947
*Fgf8*	F: CCGAGGAGGGATCTAAGGAAC R:CTTCCAAAAGTATCGGTCTCCAC	238	NM_001166361
*Gdf9*	F: TCTTAGTAGCCTTAGCTCTCAGG R: TGTCAGTCCCATCTACAGGCA	116	NM_008110
*Mfn1*	F: ATGGCAGAAACGGTATCTCCA R: CTCGGATGCTATTCGATCAAGTT	153	NM_024200
*Mfn2*	F: TGACCTGAATTGTGACAAGCTG R: AGACTGACTGCCGTATCTGGT	205	NM_133201
*Opa1*	F: CGACTTTGCCGAGGATAGCTT R: CGTTGTGAACACACTGCTCTTG	224	NM_001199177
*Padl6*	F: TGGTAGGCATGGAAATCACCT R: GACGGAGCTAGAGATGTGGAT	110	NM_153106
*Palrzd*	F: GCTGGCGACGTAGAAGAAGAC R: CTGCTTTCGATGCTCCAGAAG	111	NM_027101
*Tpx2*	F: GATGCCCCCACCGACTTTATC R: CTTGTTCTCCAAGTTGGCCTT	102	NM_001141976
*β-Actin*	F: GATGCCCCCACCGACTTTATC R: CCAGTTGGTAACAATGCCATGT	154	NM-007393

## Data Availability

The data used to support the findings of this study are available from the corresponding author upon request.

## Additional Points

*Summary Statement.* Mitochondrial Ca^2+^ ([Ca^2+^]_mt_) plays a key role in early embryonic development. In this study, we firstly demonstrated that the ERK/MAPK signaling pathway is regulated by [Ca^2+^]_mt_ in MII oocytes, of which disorder influences the epigenetic modification and preimplantation development of the mouse embryo.
